# Making America Healthy Again: Remedies for Robert F. Kennedy Jr.'s Campaign against Chronic Disease

**DOI:** 10.1002/hast.5020

**Published:** 2025-07-14

**Authors:** Lawrence O. Gostin, Alexandra Finch, Peter Lurie

**Keywords:** MAHA, MAHA Commission, RFK Jr., chronic disease, policy, bioethics

## Abstract

*Chronic diseases impose enormous health and economic burdens in the United States, especially on marginalized populations, and demand evidence‐based, equity‐focused interventions. To combat chronic disease, the Trump administration established the Make America Healthy Again Commission, chaired by Department of Health and Human Services Secretary Robert F. Kennedy Jr. However, the MAHA Commission appears to be both ideologically driven and scientifically unsound, and as a consequence, its prospects of proposing policies that meaningfully address chronic disease are exceedingly low. Instead of adopting an evidence‐based approach, in his actions and comments to date, Secretary Kennedy has undermined established science, particularly on vaccines; gutted U.S. science and public health infrastructure, including segments responsible for addressing chronic disease; and prioritized concerns that have little basis in science. This essay describes the burden of chronic diseases in the United States, digs deeper into the MAHA agenda, discusses the ethics of chronic disease prevention, and identifies evidence‐based policies that would actually be effective in combatting chronic diseases*.

## Policy & Politics

On February 13, 2025, President Trump issued an executive order establishing the Make America Healthy Again Commission, an all‐of‐government approach focused on preventing chronic disease.^1^ The commission is chaired by Department of Health and Human Services (HHS) Secretary Robert F. Kennedy Jr., who has been charged with leading the government's efforts to prevent and mitigate chronic disease. Such a commission ought to unify public health advocates. After all, chronic diseases have long been the leading cause of morbidity and premature mortality in the United States, as well as a major driver of health care costs. Low‐income and racial minority communities often bear a disproportionate burden from chronic diseases.

Yet the prospects that the MAHA Commission will propose policies that meaningfully address chronic diseases are exceedingly low. Kennedy's record and current actions are more likely to undermine evidence‐based policies. Just four months into his role as secretary, he has already cast doubt on established science, fired staff across HHS, slashed funding for research, and pursued fringe health policies while showing no signs of producing and following scientific evidence that would improve the public's health.

In this essay, we describe the burden of chronic diseases in the United States, dig deeper into the MAHA agenda and its recently released assessment,^2^ discuss the ethics of chronic disease prevention, and identify evidence‐based policies that would actually be effective in combating chronic diseases.

## America's Chronic Disease Epidemic

Let's start with an indisputable fact: America is not healthy. Americans are sicker and die younger than people in most of our peer countries do,^3^ even though, on a per capita basis, the United States spends roughly twice the amount on health services as do our peers.^4^ Cardiovascular disease remains the top cause of death, with almost 945,000 annual deaths from heart disease and stroke, while cancer accounts for over 600,000 deaths and 1.7 million new diagnoses each year.^5^ While overall cancer incidence has slowly declined since the early 1990s, cancer incidence and mortality rates among Americans under age fifty have been rising.^6^

Diabetes affects nearly one in ten Americans, with most diagnosed with type 2 diabetes, which is largely preventable.^7^ Trends among children are worrying: the incidence of pediatric type 2 diabetes nearly doubled between 2002 and 2018.^8^ And over two in five adults have metabolic syndrome—a cluster of symptoms that includes high blood sugar, high blood pressure, and high triglycerides and increases the risk of cardiovascular disease, type 2 diabetes, and stroke—and this fraction has been growing.^9^

The burden of chronic disease disproportionately affects low‐income communities, people with disabilities, people of color, and people with less formal education. For instance, Black individuals in the United States are about one‐third more likely to die from heart disease than are non‐Hispanic white individuals,^10^ and the prevalence of diabetes is significantly higher among people living in poverty compared to those with higher incomes.^11^

The primary modifiable risk factors for chronic diseases are smoking, alcohol consumption, physical inactivity, and unhealthy diets. Significant progress has been made in tobacco control, with smoking prevalence having plummeted over the past fifty‐plus years, especially among youth.^12^ Alcohol consumption has fluctuated—increasing from the 1950s through the early 1980s, then falling, and increasing again since the late 1990s.^13^ Binge drinking has increased among adults.^14^

Levels of insufficient physical activity among Americans increased by 15 percent from 2000 to 2022.^15^ Trends in obesity are especially noteworthy. Since the mid‐1970s, its prevalence has risen from 15 to 40 percent. Now, nearly three‐quarters of adults (74 percent) are estimated to be overweight or obese.^16^ The increase, in relative terms, is even greater for children, with childhood obesity rates climbing from 5 percent in 1978 to nearly 20 percent by 2020.^17^

The standard American diet is a major culprit. The average American consumes almost 58 percent of their total dietary energy intake from ultraprocessed foods,^18^ which are often high in saturated fats, sodium, and added sugars and low in essential nutrients and dietary fiber. However, an individual's ability to modify dietary risk factors is constrained by social and environmental factors, including industry marketing, confusing labeling and packaging, the lack of locally available fresh foods, and cost.

Other types of environmental factors also increase individuals’ risk of developing chronic diseases. Air pollution, for example, is a major contributor to stroke, heart disease, respiratory disease, and lung cancer.^19^

Beyond health impacts, chronic diseases, including mental illness, impose significant social and economic costs. Chronic conditions account for 90 percent of the nation's $4.5 trillion annual health care expenditure and impose additional indirect costs resulting from reduced productivity, absenteeism, and premature mortality.^20^ Taken together, these costs can result in catastrophic health expenditures for individuals and families, exacerbating poverty and health disparities in vulnerable populations. Medical debt, meanwhile, is a leading cause of bankruptcy.^21^

## False Claims and a Flawed Approach

The MAHA Commission's mission is “to aggressively combat” critical health challenges facing Americans, including “obesity, diabetes, and other chronic diseases.”^22^ It is tasked with addressing the threat posed by “over‐utilization of medication, certain food ingredients, [and] … certain other exposures” including “selective serotonin reuptake inhibitors, antipsychotics, mood stabilizers, stimulants, and weight‐loss drugs.” The commission is thus seemingly designed to target the long‐held pet peeves of the secretary, such as those related to synthetic food dyes. While food dyes serve no nutritional function, they are not understood to be a significant driver of chronic disease.^23^ But on April 22, Kennedy announced the intent of the U.S. Food and Drug Administration and HHS to phase out synthetic food dyes from the food supply by the end of 2026 through voluntary action by the food companies.^24^

Kennedy—long opposed to fluoridation of the water supply—plans to order the Centers for Disease Control and Prevention (CDC) to stop recommending fluoride in drinking water,^25^ and the FDA is now proposing to remove ingestible fluoride prescription drugs from the market.^26^ As a result of his advocacy, Utah and Florida banned water fluoridation,^27^ and many red states are likely to follow. Water fluoridation is regarded as a highly effective public health measure to combat tooth decay and a cost‐effective strategy for lower‐income communities that face the greatest burdens of chronic dental disease.^28^

Kennedy has also long pushed the debunked theory that measles‐mumps‐rubella vaccines cause autism. On April 16, Kennedy drew the ire of mental health professionals and parents by making disparaging remarks about the capacities of children with autism, which the American Autism Society called “harmful, misleading, and unrealistic.”^29^ On the same day that Kennedy's own agency ascribed rising autism rates to improved screening and awareness,^30^ the secretary declared, “‘Genes don't cause epidemics’ … . ‘You need an environmental toxin.’”^31^ Kennedy has appointed David Geier, a known vaccine skeptic who has spread false claims about vaccines and autism, to lead a critical study on vaccine safety, even though Geier has been sanctioned by the Maryland Board of Physicians for practicing medicine without a license.^32^

Remarkably, the MAHA Commission's founding executive order purports to “study the scope of the childhood chronic disease crisis and any potential contributing causes,”^33^ yet it neglects decades of scientific consensus on the primary drivers of chronic disease, including excessive consumption of fats, sugars, sodium, tobacco, and alcohol.^34^ All of these go unmentioned in the order.

On May 22, the MAHA Commission released its initial assessment identifying what it considered to be the key drivers of the childhood chronic disease crisis.^35^ The report largely focuses on ultraprocessed foods, environmental chemicals, and “overmedicalization,” particularly relating to medicines like antidepressants, stimulants, glucagon‐like peptide‐1 (GLP‐1) drugs, and gender‐affirming treatments. It further questions the childhood vaccination schedule and its expansion since 1986, claiming that there had been “limited scientific inquiry into the links between vaccines and chronic disease” as a basis for further inquiry. (Notably, on June 9, Kennedy removed all 17 members of CDC's Advisory Committee on Immunization Practices, which makes recommendations to CDC on the vaccination schedule).^36^ While the MAHA Commission report calls for “gold‐standard scientific research,” it also includes several fake and inaccurate citations to scientific studies, drawing concerns surrounding the use of artificial intelligence in the report's drafting.^37^ It is especially concerning that a report based in part on false information is expected to inform policy decisions regarding children's health.

The interactions between chronic and infectious diseases are real and complex. Chronic conditions can heighten an individual's vulnerability to infectious diseases, as demonstrated during the Covid‐19 pandemic, and infectious pathogens like the human papillomavirus and hepatitis B and C viruses are significant causes of cancer.^38^ The MAHA Commission's chronic disease focus should not undermine infectious disease prevention and treatment, especially amid ongoing H5N1 influenza and measles outbreaks. Yet, as a presidential candidate and during confirmation hearings, Kennedy said that he would order a “break” on infectious disease research for eight years.^39^ He amplified misinformation on infectious diseases, including questioning the relationship between HIV and AIDS.^40^

Under Kennedy's authority, government funding and programs to study chronic diseases have already been slashed. The Diabetes Prevention Program, which had been studying type 2 diabetes prevention since 1996, lost its government funding and was forced to end abruptly.^41^ HHS also canceled funding for research on minority health and health disparities—identifying programs to be cut by searching for key terms related to diversity, equity, and inclusion and vaccine hesitancy.^42^ Ongoing lawsuits contest the legality of these cuts.^43^

If Kennedy were serious about addressing chronic diseases, he would bolster health agencies, not dismantle them. Yet on March 27, HHS announced a sweeping restructure, including a 10,000‐employee reduction,^44^ which is to be on top of the 10,000‐employee reduction attributable to terminations and retirements from January 28, 2025, through February 2025, in line with President Trump's “government efficiency” executive order.^45^ The mass layoffs have already gutted critical offices addressing chronic diseases, including the FDA's Center for Tobacco Products and the CDC's Office of Smoking and Health.^46^ The job cuts were part of a major reorganization, ending important agencies such as the Substance Abuse and Mental Health Services Administration and the National Institute for Occupational Safety and Health while creating a new agency within HSS called the Administration for a Healthy America.

## The Ethics of Chronic Disease Prevention

There are compelling ethical reasons supporting the implementation of effective interventions to prevent and manage chronic diseases. Living healthier without debilitating chronic disease offers reduced pain and suffering, promotes independence, yields cost savings, and offers benefits in longevity and quality of life. At the population level, reducing chronic diseases lowers health care costs, promotes productivity at school and work, and eases social and economic strain on families and communities.

To some, government action concerning diet, physical activity, tobacco, and alcohol infringes on individuals’ autonomy and free will and is paternalistic (the work of the “Nanny State”). This argument is shortsighted because health itself has benefits and the costs of ill health are often externalized and borne by society. But most evidence‐based policies addressing chronic diseases (including those we discuss later) do not compel people to do, or not to do, certain things; instead, they provide incentives and disincentives (“nudges”) or structure commercial, built, or informational environments to encourage healthier choices.^47^

Individual choices are already subject to powerful influences in a decidedly unhealthy direction. Industry marketing, product placement in supermarkets, false or misleading food and beverage package labeling, and the manufacturing of addictive tobacco products, alcohol, and foods high in sugar, salt, and unhealthy fats create all the wrong incentives. Individuals cannot simply improve the quality of the air they breathe, control the level of nicotine in cigarettes, or change the ingredients in most foods on store shelves. These factors are modifiable only through policy and regulation. Evidence can guide policy‐makers about which activities promote health and which do not—and which interventions are more effective at advancing population health outcomes.

Smart interventions to reduce chronic diseases advance another ethical value—justice. Given the massive and ongoing health disparities in the United States, policy‐makers have a duty to focus on the most disadvantaged and marginalized populations. That requires not only more equitable distribution of resources but also policies that are most likely to reduce crushing health inequalities.

## A Reform Agenda Backed by Evidence

If tethered to effective interventions, Kennedy's pledge to “Make America Healthy Again” would present a rare opportunity for bipartisan policy to create the conditions for healthier lifestyles. But success depends fundamentally on the secretary's embracing a rigorous, evidence‐based public health approach to chronic disease and eschewing pseudoscience and profiteering by wellness companies and influencers. We present a three‐pronged approach that holds considerable promise to reduce chronic illness.

***Adopt evidence‐based policies.*** Too often, people are blamed for their own chronic conditions. What and how much someone eats, how frequently they exercise, and their use or avoidance of tobacco and alcohol are viewed as personal choices. But chronic diseases are a predictable consequence of social, environmental, and commercial determinants that promote unhealthy lifestyles. Here are important evidence‐based policies the MAHA Commission could prioritize, though this is by no means a comprehensive list.

First, we need better tobacco control. Cigarette smoking is a leading cause of disability related to chronic disease, and it results in massive health care and economic costs.^48^ Yet, as noted, tobacco control is not listed among the MAHA Commission's priorities. The United States has already made major progress in reducing smoking rates, but many public health leaders have called for “endgame” strategies that would push smoking rates to negligible levels.^49^

A powerful nicotine strategy is already under consideration. Days before President Biden left office, the FDA proposed a rule to reduce nicotine in cigarettes and other tobacco products to minimal or nonaddictive levels—0.7 milligrams of nicotine per gram of tobacco, compared to the current levels of about 10 to 12 milligrams.^50^ The commission should call for the FDA to finalize this rule, but the prospects for such action remain low. Consider that, soon after taking office, President Trump withdrew an FDA proposed rule to ban menthol cigarettes, even though, compared to nonmenthol cigarettes, they are considered harder to quit, they lure young people to smoking, and their use disproportionately impacts Black individuals.

The commission could also propose federal legislation setting a generational cutoff for tobacco purchases (smoke‐free generation laws)—banning the sale of tobacco to anyone born after a certain date—phasing out tobacco sales and protecting expanding generations from tobacco across the life course. New Zealand became the first country to promulgate a smoke‐free generation law in 2022, but it was reversed in 2024. The United Kingdom also passed a similar bill that has not yet become law.^51^ At the least, the commission could propose raising the legal age for purchasing tobacco products from twenty‐one to twenty‐five, which could help reduce the number of young people who initiate smoking—a priority because most smokers begin smoking well before age twenty‐five.^52^

Second, we need to limit the consumption of foods high in saturated fat, sugar, and sodium. Kennedy has emphasized the importance of dietary contributors to chronic disease. He has pledged to ban ultraprocessed foods from school cafeterias and asked the Department of Agriculture to ban the purchase of sugar‐sweetened beverages (SSBs) with Supplemental Nutrition Assistance Program (SNAP) benefits. Ultraprocessed foods have been associated with over thirty health conditions in observational studies,^53^ while SSBs increase the risk of diabetes, cardiovascular disease, and dental caries. Although reducing harmful ultraprocessed foods in school meals is a worthy undertaking, the value of banning the purchase of SSBs with SNAP benefits is questionable. SNAP beneficiaries should have agency in their food purchases, and, in any case, they could still buy SSBs with their own funds.

Here are some better ideas:

- **SSB taxes.** Studies in the United Kingdom, Mexico, Saudi Arabia, and South Africa have demonstrated that taxes on SSBs, when applied at a national level, are effective in reducing consumption of sugars.^54^
- **Front‐of‐pack food labels.** Nutritional labeling is confusing and often disregarded by consumers, with variations in use of this information by income level and educational attainment.^55^ The FDA should require interpretive front‐of‐pack labeling, such as those that display prominent warning labels stating that a food is “high in” a particular harmful nutrient. In Chile, such labels reduced the sugar content of purchases^56^ and prompted companies to reformulate their products to contain less of the targeted nutrients.^57^ The FDA has proposed such a labeling system,^58^ which the MAHA Commission would do well to endorse.
- **Restrictions on the marketing of certain foods.** Evidence indicates that restrictions on marketing (which influences purchasing of food products) are highly cost effective.^59^ The MAHA Commission should propose restrictions on marketing of foods with high levels of sugar, sodium, and saturated fats. This will have to be consistent with the U.S. Supreme Court's commercial speech doctrine, which recognizes commercial speech as a protected form of speech, except in cases of false or misleading commercial speech.^60^ The commission could start by protecting children from ads marketing foods known to be harmful as healthy.
- **Sodium reduction.** The MAHA Commission should also recommend finalizing the FDA's proposed medium‐term sodium‐reduction targets to reduce the consumption of sodium across the food supply, as such consumption is linked to increases in blood pressure—a major risk factor for heart disease.^61^
- **Better access to weight‐loss medications.** Even with strong preventative measures targeting SSBs and ultraprocessed foods, many people with overweight and obesity will benefit from clinical interventions like GLP‐1 receptor agonists to effectively manage their condition. These medications have been shown to be effective in reducing weight, type 2 diabetes, cardiovascular diseases, and total mortality.^62^ However, HHS recently dropped Biden‐era plans for Medicare and Medicaid to cover GLP‐1 agonists for certain patients. Kennedy has criticized the use of GLP‐1s (they also come under scrutiny in the recent MAHA assessment report), but the MAHA Commission should expand equitable access to these medications.
- **Clean air.** The MAHA Commission identified disparities between the United States and its peer nations in rates of cancer and asthma but did not identify air pollution as a target for intervention. To reduce the incidence of asthma and cancer, the commission should oppose the president's decision to roll back Environmental Protection Agency regulations, including limits on pollution from tailpipes and smokestacks.^63^ Additional policies that support walking and cycling infrastructure to reduce air pollution from traffic would also benefit health by promoting physical activity.

***Reduce health inequities.*** The MAHA Commission, if it adhered to the evidence, would find that the root causes of chronic disease lie not in the presence of food dyes, but in systemic inequities that result in unequal access to healthy foods, green spaces, and preventive health services.

Eighteen million people in the United States are food insecure, and 6.1 percent of the population has limited access to a food store.^64^ While Kennedy highlighted health disparities between the United States and peer nations, he neglected to mention even starker domestic disparities, with a 14.6‐year life expectancy gap between American men in the top 1 percent and the bottom 30 percent of income.^65^ But, again, the administration's policies cut against sensible reforms. Consider its crusade against diversity, equity, and inclusion policies; its tariffs on imports of fresh produce; and its proposals to make substantial cuts to SNAP benefits.^66^ Equitable access to health services could help prevent and treat chronic diseases at an earlier, manageable stage. Yet the administration plans major cuts to clinical programs that serve poor individuals, such as Medicaid and the Children's Health Insurance Program.^67^

***Trust in science and public health agencies.*** Public health does not work without public trust. Patients are unlikely to accept vaccines, follow medical advice, or seek treatment or diagnosis if they do not trust science. They are more likely to disregard public health guidance about nutrition and physical activity if they distrust the data or if health leaders undermine evidence. The MAHA movement could reach its ardent supporters with messages of trust in science and public health, but it is doing the opposite.

Promising “radical transparency,” the secretary has provided anything but. The MAHA Commission's inaugural meeting was held behind closed doors,^68^ and Kennedy laid off staff who handle agency communications and Freedom of Information Act requests. Kennedy has canceled scientific advisory committee meetings and fired all the members of CDC's vaccine advisory committee, falsely claiming that its members have widespread conflicts of interest.^69^ And his push for greater transparency on vaccine safety is an unmistakable attempt to propagate and legitimize his own vaccine misinformation. For MAHA to succeed, it should be guided by evidence and processes that reflect the ideals of transparency that Kennedy says he espouses.

## Barriers to Making America Healthy

Ultimately, the barriers to the administration's stated aims are of its own creation. The MAHA Commission can succeed in its goal of aggressively combating chronic disease only if its policies target the known drivers of chronic disease and are firmly grounded in ethics and scientific evidence. And those policies can be monitored, implemented, and improved only if the health and scientific institutions that underpin them are not completely decimated.

With the popularity of the MAHA agenda among some of its supporters, the administration has a rare opportunity to drum up bipartisan support for health promotion. Yet the actions and ideology of Secretary Kennedy and the Trump administration more broadly are further undermining public trust and hence our ability to combat chronic disease and improve America's health.

## Acknowledgment

Peter Lurie's work on this project was funded by the Harvey Motulsky and Lisa Norton‐Motulsky fund.
